# Auscultation Serendipitously Leading to the Discovery of a Type A Aortic Dissection: The Lost Art of Physical Examination

**DOI:** 10.7759/cureus.5358

**Published:** 2019-08-10

**Authors:** Ahmed Elzanaty, Ebrahim Sabbagh, Dinkar Kaw, Ehab Eltahawy

**Affiliations:** 1 Internal Medicine, University of Toledo Medical Center, Toledo, USA; 2 Cardiology, University of Toledo Medical Center, Toledo, USA; 3 Internal Medicine: Nephrology, University of Toledo Medical Center, Toledo, USA

**Keywords:** aortic dissection

## Abstract

In the current era of rapidly advancing and evolving medicine, a huge emphasis has been placed on the utilization of diagnostic tests and imaging, as well as on following updated guidelines, at the expense of focusing on clinical exams and honing these skills. Poor physical exam skills is a definite risk to patient safety, as they might lead to the misdiagnosis of, sometimes, fatal diseases. In this case, we discuss a case of complicated aortic dissection type A that initially presented to us as a case of worsening chronic graft rejection. Aortic dissection type A was solely uncovered by clinical exam despite unusual presenting symptoms and grossly benign basic labs and initial chest radiograph.

## Introduction

Aortic dissection is a rare but catastrophic disease, which classically presents with severe chest and/or abdominal pain that, at times, radiates to the back, with the occasional association of symptoms of end-organ ischemia. Populations who are at a higher than usual risk for aortic dissection include those suffering from hypertension, collagen disease, history of aortic surgery, trauma, pregnancy, chronic kidney disease [1-2], or vasculitis, among others. There are few case reports that link type A aortic dissection to either patients with renal transplant [2-3] or systemic lupus erythematosus (SLE) [4-5]. In our case, we will discuss how the collaboration of a multidisciplinary team led to the discovery of an unusual presentation of complicated aortic dissection.

## Case presentation

A 35-year-old lady with a known history of controlled hypertension, avascular necrosis of the right hip, lupus nephritis status post cadaveric renal transplant in 2014, with chronic graft rejection, and a history of abdominal pain that was attributed to an abdominal wall hematoma diagnosed previously by ultrasound (US) abdomen, presented to an outside facility with severe 10/10 stabbing chest pain radiating to the back that started suddenly while the patient was sitting down; she also complained of some abdominal pain that was mild as compared to her chest pain. At the outside facility, the patient was noted to be hypertensive. Serial electrocardiograms (EKGs) and troponins were done, and they were unremarkable. The patient was later found to have acute kidney injury with a creatinine of 7.4 and her last known creatinine was around 4.2. Given the patient’s acute kidney injury, computed tomography (CT) abdomen without contrast was done to evaluate the renal graft, which eventually came back unremarkable. The patient was then transferred to our facility due to the availability of transplant services in our hospital with a working diagnosis of acute on top of chronic kidney disease secondary to worsening chronic graft rejection.

Upon initial presentation to our hospital, the patient had a blood pressure reading of 214/89 as well as an episode of chest pain. The patient's pain and blood pressure slowly improved after receiving 1 mg of hydromorphone hydrochloride, with blood pressure returning to 130/70 mmHg. The patient's home blood pressure medications were then resumed.

From that point onward, the patient was no longer complaining of any chest or abdominal pain. Her blood pressure ranged around 113/70 throughout her stay. Upon questioning the patient about her blood pressure control, she said that it was always controlled and that it was only high on presentation because of the severe pain she was in. A complete physical exam was done on admission, including bilateral measurements of blood pressure in both arms that did not show any discrepancy. Given the patient's stable clinical condition and lack of any pain, the possibility of aortic dissection was kept low on the differential diagnoses list, and we attributed the chest pain to possible acute coronary syndrome vs pericarditis. The plan then was to trend troponin with serial EKGs and a routine transthoracic echocardiogram. Meanwhile, the nephrology team was consulted and during their examination, they appreciated a possible friction rub that was missed by the primary team, and the priority of the echocardiogram was changed to stat to rule out possible uremic pericarditis. Bedside echocardiogram done by the cardiologist revealed new moderate aortic valve regurgitation, with mild aortic dilatation and possible aortic dissection, as well as mild to moderate pericardial effusion. Given the urgency of the situation, the patient was started immediately on esmolol drip and was transferred to the critical care unit after consulting the cardiothoracic surgeon. The patient had a trialysis catheter inserted emergently in preparation for hemodialysis, and she went for CT angiography. The CT findings reported a type A aortic dissection extending from the aortic root to the abdominal aorta and extended through the left common carotid artery and celiac trunk. It also showed the intimal flap extending into the origin of the left main coronary artery. In addition, an area of rupture with pseudoaneurysm and extravasation of contrast along the ascending aorta left wall was seen, which was causing the moderate to large hemopericardium.

The patient was started immediately on esmolol drip to control her heart rate, as her blood pressure was already within the target range. She also got an emergent hemodialysis session and was scheduled for an emergent vascular surgery on the same day.

Pertinent investigations upon initial presentation

● Troponin I: 0.03 on admission with a minimal rise to 0.06 after three hours

● Creatinine: 7.4 mg/dl at the outside facility, which increased to 8.33 mg/dl

● Bicarbonate: 13 meq/L

● Anti-double stranded DNA (anti-dsDNA): <1/10 normal

● C3 & C4: 57 (lower limit of normal is 79) and 16 (normal)

● Erythrocyte sedimentation rate (ESR): 36

● Potassium: 5.5 meq/L

● EKG: nonspecific ST changes

**Figure 1:**
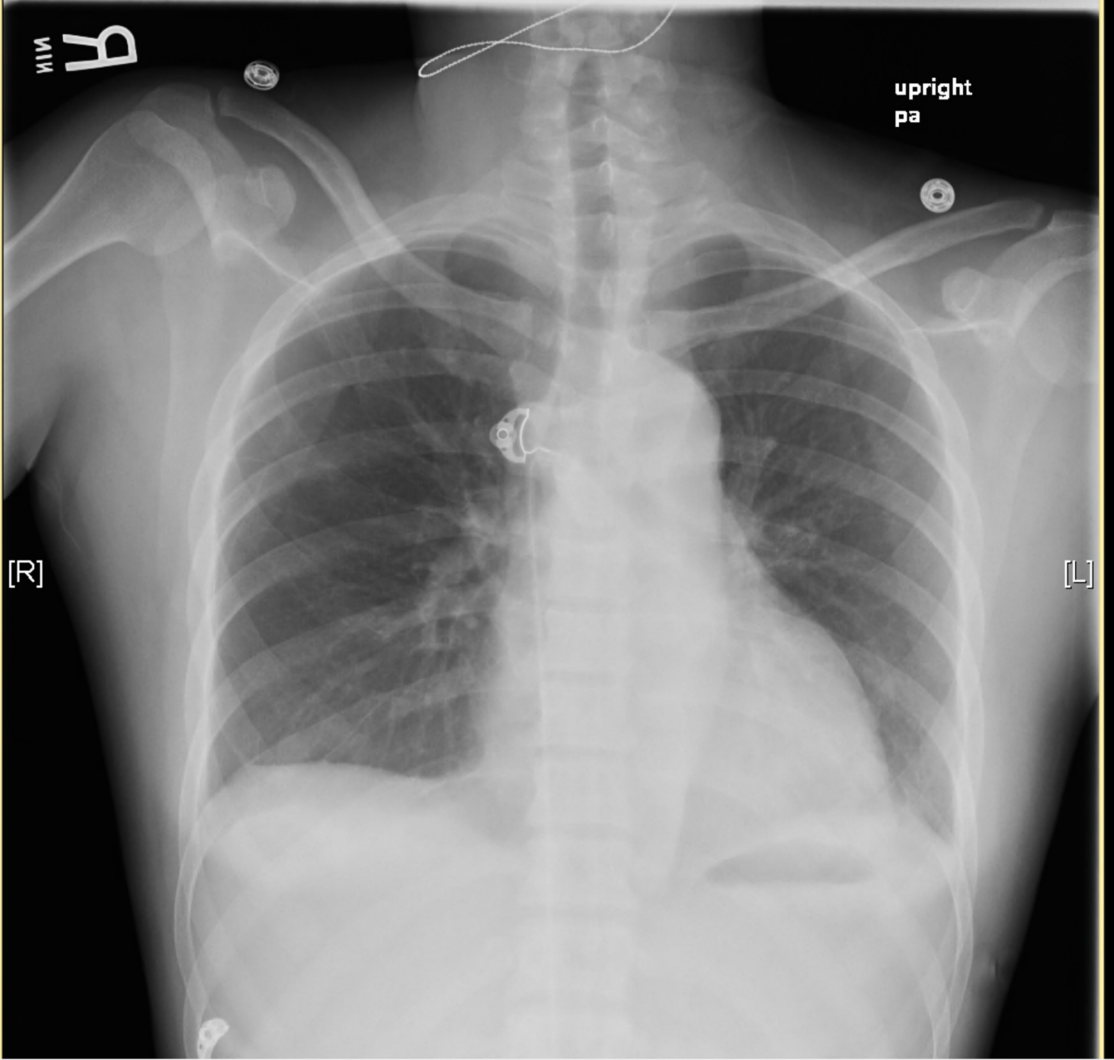
Chest X-ray upon presentation showing normal mediastinum

● Chest X-ray (XR): No widening mediastinum, with no radiographic evidence of acute cardiopulmonary process (Figure 1)

**Figure 2:**
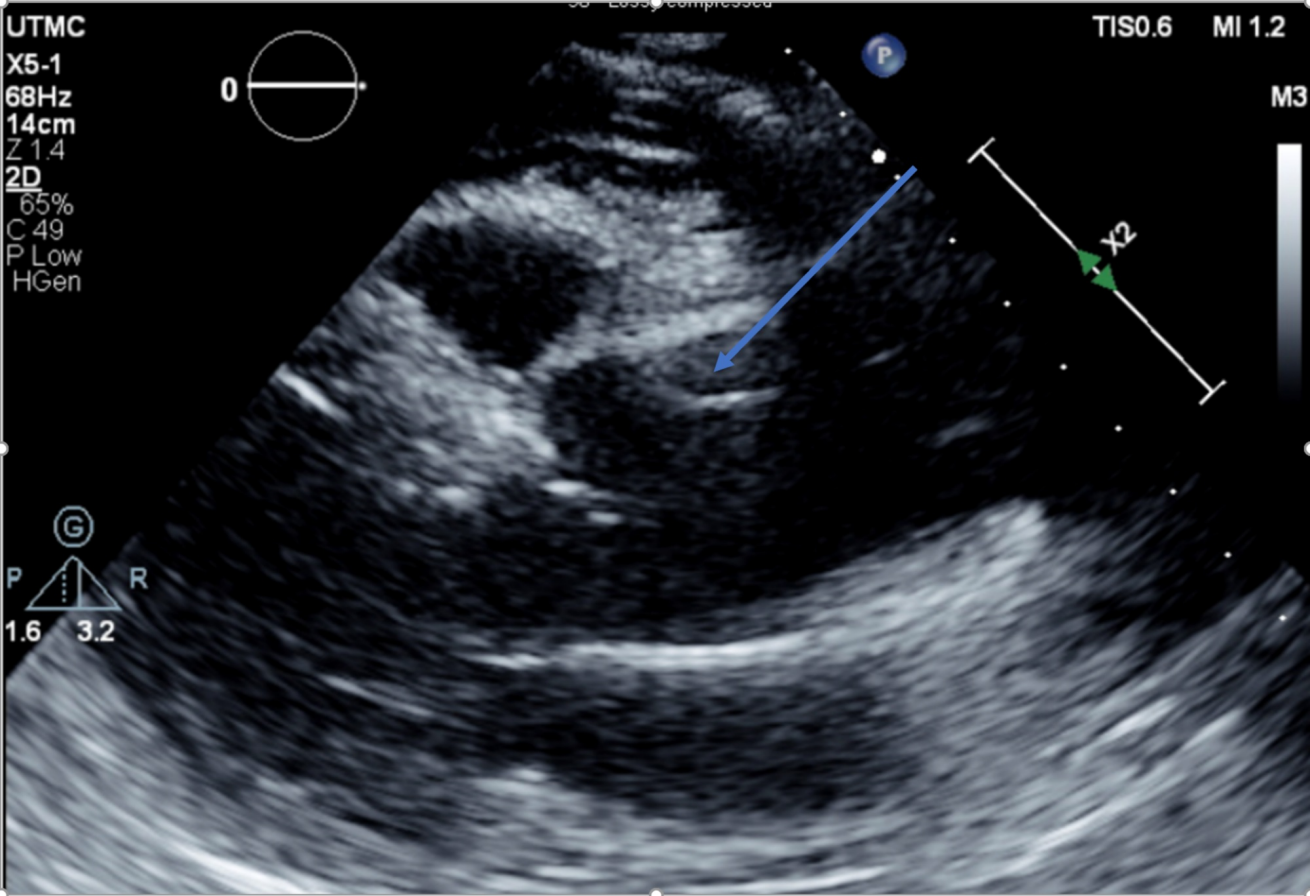
Transthoracic echocardiogram picture showing aortic flap

● Transthoracic echocardiogram after the suspect pericardial rub: Global left ventricular systolic function is hyperdynamic. There were moderate left ventricular hypertrophy and moderate aortic valve regurgitation. A dissection of the aortic root is seen. There were also mild aortic dilatation and a small to moderate pericardial effusion. Echodense material within the pericardial space suggested a clot or fibrinous elements (Figure 2).

**Figure 3:**
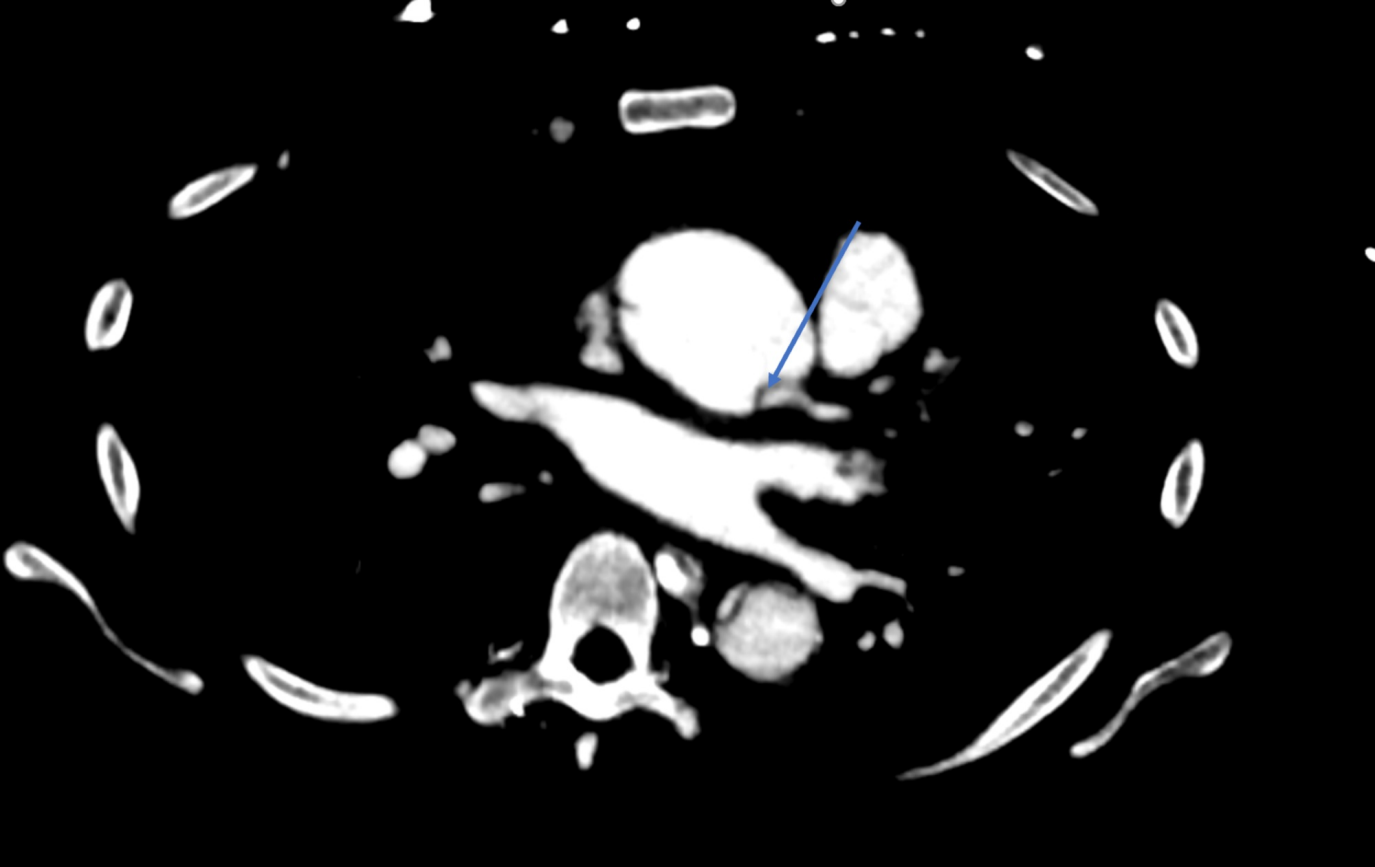
CT scan showing extension of dissection to the coronaries

**Figure 4:**
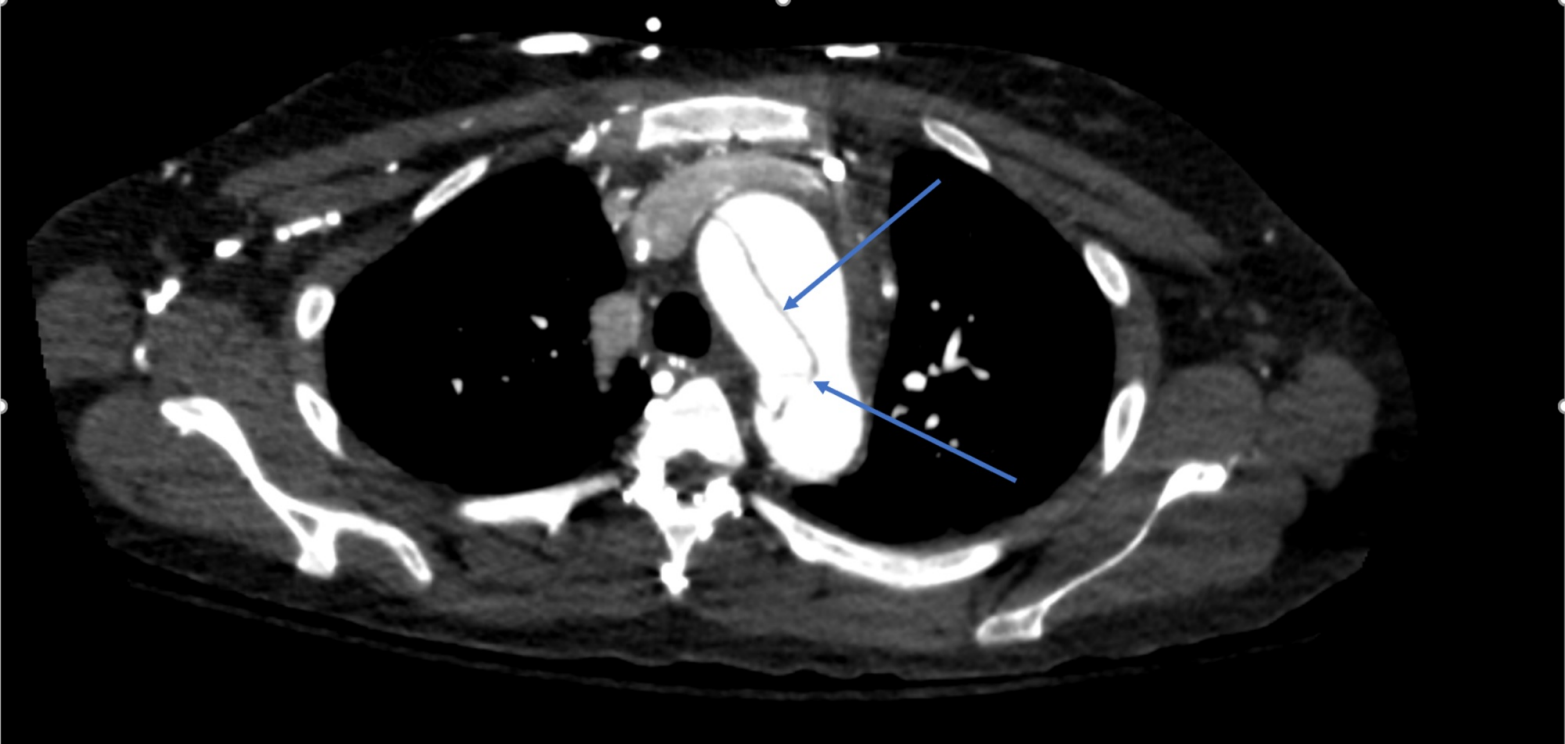
CT scan showing aortic dissection

**Figure 5:**
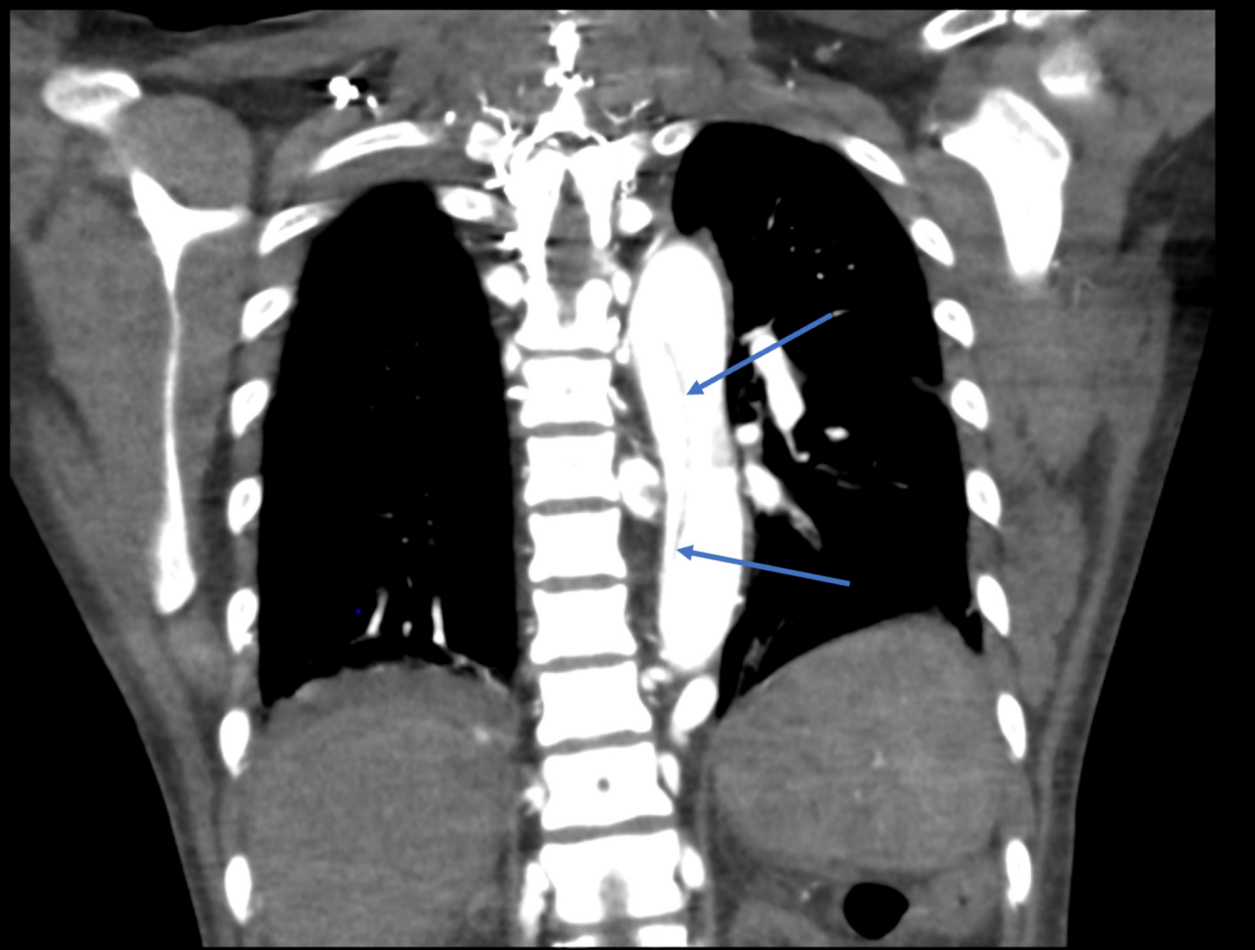
CT chest scan, coronal view, showing aortic dissection

● CT angiography (CTA) chest done after confirming an acute aortic regurge on echocardiography: There was a type A aortic dissection extending from the aortic root to the abdominal aorta, which involved the celiac trunk. The ascending aorta was dilated, measuring 4.2 cm. The intimal flap extended down to the aortic valve plane and extended into the origin of the left main coronary artery. There was also an area rupture with a pseudoaneurysm along the left wall of the ascending aorta, which could account for the large amount of hemopericardium present. Contrast was present in the true and false lumen. The dissection extended to the left common carotid artery with a good enhancement of the main three branches of the aortic arch (Figures 3-5).

Outcome and follow-up

The patient had a successful surgery with a median sternotomy and repair of type A aortic dissection, with a 29-mm Gelweave graft (Vascutek, Inchinnan, UK) and a complex resuspension of the aortic valve. The patient was successfully extubated. She was initially maintained on continuous venovenous hemodialysis but then later transitioned to an intermittent hemodialysis regimen. The patient made a good recovery, and she was later discharged with close outpatient follow-up.

## Discussion

Aortic dissection is a fairly uncommon disease, with a grave consequence if not detected and managed early, with an incident of 2.6 to 3.5 per 100,000 person-years and an in-hospital mortality of around 27.4% [6-7], with mortality approaching 1% per hour for the first 48 hours in patients with a Stanford type I dissection [8]. Most of the mortalities are caused by either rupture of the dissection into the pericardium with tamponade formation or rupture into the aortic annulus, leading to severe aortic regurge, obstruction of the coronary artery ostia leading to myocardial infarction, and end-organ failure due to abdominal aortic branch vessel obstruction [9-10].

To make the diagnosis of aortic dissection, a high suspicion of the disease should be kept in mind, especially with the presence of the high-risk clinical triad: acute sharp chest/abdominal pain, a variation in pulse and/or blood pressure, and mediastinal widening on chest radiograph [11]. If those high-risk features are there, confirming the diagnosis with advanced imaging is required. Usually, CT angiography is the test of choice due to its availability and ease of use [12]; MR angiography, as well as transoesophageal echo, is also a valid alternative [13]. It is worth mentioning that both CT and MR angiography are of high risk in patients with chronic kidney disease as the contrast used might tip them into the end-stage renal disease (ESRD) category or causing grave complications like nephrogenic systemic fibrosis [14] while the transesophageal echocardiography (TEE) is operator dependent and requires a certain level of expertise to interpret the findings.

Our patient was at a higher risk than the general population for developing aortic dissection with her SLE and chronic kidney disease. She was also suffering from a failing renal graft, which made the possibility of the patient being transitioned from the ‘chronic kidney disease’ category to ‘end-stage renal disease,’ requiring long-term hemodialysis, after receiving the contrast for the imaging extremely likely, and this is the most probable reason why physicians at the other facility as well as the admitting team to our facility weren’t so keen on exposing the patient to contrast despite the patient presenting with chest pain typical of dissection accompanied by elevated blood pressure.

In our case, the patient’s diagnosis would have been missed if not for the excellent teamwork and communication between the different multidisciplinary teams, with the nephrologist hearing a rub and then making arrangements to expedite the transthoracic echo, which was done immediately by the cardiologist who detected the dissection.

## Conclusions

Aortic dissection is a rare but fatal disease and requires a high degree of suspicion. Although the hallmark features of the disease are severe chest pain with a possible pulsus deficit or an unequal blood pressure reading on different arms. It can sometimes present silently. That is why, in difficult scenarios with a high probability of the disease alongside a high risk of complications from investigations (namely, contrast exposure in patients with chronic kidney disease), a thorough clinical exam, alongside a multidisciplinary team discussion, is warranted to guide the next appropriate mode of action.
